# Individual Differences in the Affective Experience of Writing a Gratitude Letter: Who Benefits Most?

**DOI:** 10.3390/bs16020232

**Published:** 2026-02-05

**Authors:** Tanya K. Vannoy, Lisa C. Walsh, Luke Liao, Sonja Lyubomirsky

**Affiliations:** 1 Department of Psychology, University of California, Riverside, Riverside, CA 92521, USAsonja@ucr.edu (S.L.); 2 Department of Psychology, Nanyang Technological University, Singapore 639798, Singapore; lisa.walsh@ntu.edu.sg

**Keywords:** gratitude, gratitude letter, well-being, person–activity fit, individual differences, personal growth

## Abstract

This study merged archival data from three separate experiments to investigate the typology of individuals who benefit most and least from gratitude letter writing interventions (*N* = 487). First, k-means clustering of pre- to post-intervention changes in affect revealed three distinct groups: Buffered, Mixed Feelings, and Backfired. The Buffered cluster comprised individuals who, on average, experienced decreases in negative affect (e.g., less frustration) but no changes in positive emotions (e.g., joyful). The Mixed Feelings cluster experienced increases in positive affect, alongside self-conscious emotions, particularly indebtedness, which became more closely aligned with uplifting emotional states following the intervention. The Backfired cluster experienced decreases in positive feelings and increases in negative affect. Next, differences in individual characteristics across clusters indicated that those in the Buffered cluster were relatively more neurotic, had higher baseline negative feelings, and lower trait gratitude. Individuals in the Mixed Feelings cluster tended to be more dispositionally grateful and seemed to invest more effort into the activity. Finally, individuals in the Backfired cluster were also relatively more grateful and had higher baseline positive affect. These findings contribute to understanding individual differences in the effectiveness of gratitude letter interventions and highlight opportunities to tailor such activities to promote personal growth.

## 1. Introduction

Gratitude, considered both a trait and emotional state (Emmons & McCullough, 2003), involves recognizing and appreciating the positive aspects of one’s life while acknowledging that these benefits often stem from the intentional actions of others (Emmons & McCullough, 2003). As an emotion, gratitude broadens thoughts and actions that help build personal and interpersonal resources (Fredrickson, 2004), encouraging individuals to reflect on benefits received—freely given by others—and fostering a desire to reciprocate kindness. These feelings, in turn, reinforce social bonds and contribute to a cycle of positive emotions and connection. Over time, this cycle fosters personal growth and a sense of belonging, thereby enhancing well-being (Fredrickson, 2004; Kashdan et al., 2009).

Compared to less grateful individuals, those high in trait gratitude report greater subjective well-being (e.g., life satisfaction and positive affect) and stronger prosocial feelings (e.g., empathy), and lower levels of envy, depression, and anxiety (McCullough et al., 2002). Gratitude also enhances trust (Dunn & Schweitzer, 2005), social connections, and prosocial behaviors such as direct and upstream reciprocity (i.e., paying it forward), thus creating a continuous loop in which one values and feels valued by others (e.g., Bono & Sender, 2018; Fredrickson, 1998; Froh et al., 2010; Grant & Gino, 2010).

Gratitude can also be cultivated. Studies testing the effects of gratitude interventions have found improvements in feelings of well-being (e.g., Armenta et al., 2022; Dickens, 2017; Oriol et al., 2020), social connection, and social support (Grant & Gino, 2010; Walsh et al., 2022b). These interventions can also buffer against stress (Reckart et al., 2017), depression, envy, and materialism (Chaplin et al., 2018; Froh et al., 2011). For example, a longitudinal study using structural equation modeling to assess directionality between gratitude, social support, stress, and depression found that, in the best direct model, practicing gratitude led to increases in feelings of social support and decreases in feelings of stress and depression over a 3-month period, controlling for personality traits (Wood et al., 2008a, Study 2).

Gratitude expressions can vary by target (social vs. nonsocial), format (lists vs. letters), and medium (e.g., letters, vs. social media posts, and texts). The present study focuses on interventions that involve writing gratitude letters. This intervention was first published by Seligman and his colleagues in 2005 (Seligman et al., 2005) as a “gratitude visit,” in which participants wrote and delivered a letter thanking someone who had been especially kind to them. That study reported increases in well-being for the letter writers (λ^2^ = 0.49 at post-test). Since then, many studies have replicated and extended these findings, distinguishing between the effects of writing a gratitude letter (e.g., Armenta et al., 2022; Lyubomirsky et al., 2011; Regan et al., 2022) and delivering the letter to the intended recipient (Kumar & Epley, 2018; Walsh et al., 2022b, 2022c).

Meta-analytic and systematic reviews of gratitude interventions suggest that average effects on well-being are small to moderate and highly variable across studies and outcomes. A recent systematic review of the effectiveness of happiness strategies found that writing gratitude letters can increase positive affect based on two pre-registered and sufficiently powered studies (Folk & Dunn, 2023). However, the same review also highlighted other sufficiently powered studies with null or mixed results. Further, a series of meta-analyses assessing the effectiveness of gratitude interventions found small-to-medium effects (using Cohen’s standards) of gratitude interventions on well-being, alongside substantial heterogeneity in effect sizes across samples and intervention formats, suggesting that gratitude letter interventions may not be equally beneficial for everyone or in all contexts (Dickens, 2017). Some studies have found that expressing or recalling gratitude can lead to simultaneous pleasant and unpleasant feelings, including elevation (e.g., moved, uplifted), connectedness, indebtedness, and guilt (Layous et al., 2017). Other studies have also reported increases in negative self-conscious emotions (Tangney, 2012), such as guilt, shame, embarrassment, and indebtedness (Armenta et al., 2022; Regan et al., 2022; Walsh et al., 2022c).

This variability in outcomes raises an important question: Are there individual characteristics or baseline affective states or traits that help explain who is more likely to experience affective improvements, declines, mixed feelings, or no change after writing a gratitude letter? Most prior work has relied on mean-level analyses that implicitly assume relatively homogeneous responses to gratitude activities. These approaches may obscure meaningful subgroups of individuals who respond differently to the same intervention. Person-centered analytic approaches, such as clustering, offer a complementary strategy by identifying distinct patterns of affective change within the same activity, thereby providing greater resolution into individual differences in intervention response.

Building on this literature, the present study adopts a clustering approach to examine whether writing a gratitude letter produces distinct affective response profiles and whether these profiles differ systematically in person characteristics.

### 1.1. Writing Gratitude Letters: The Role of Person–Activity Fit

**Figure 1:**
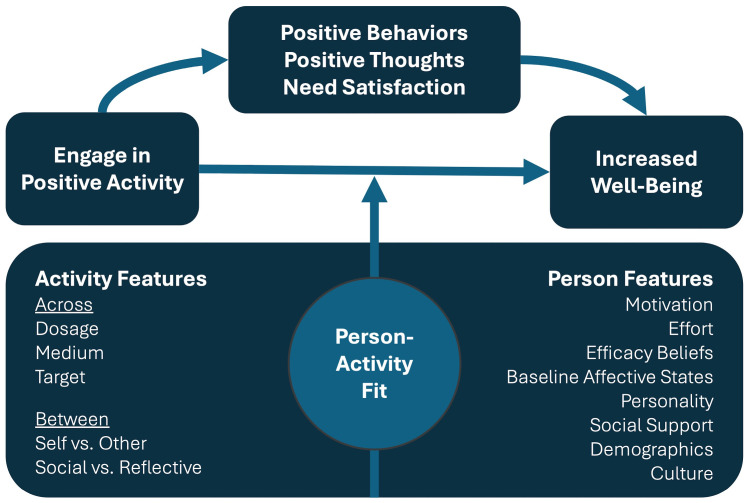
Positive Activity Model (Lyubomirsky & Layous, 2013).

Like prescription medication, the effects of writing a gratitude letter may vary depending on individual and contextual factors. Drawing from theory and empirical research, Lyubomirsky and Layous (2013) developed the Positive Activity Model (see Figure 1), which posits that the success of positive activity interventions (PAIs) depends on both activity and person features. Activity features include aspects of the PAI itself, such as the type of gratitude intervention or how frequently the activity is performed. Person features are concerned with the background, preferences, and performance of the individual practicing the PAI, such as their demographic characteristics, personality, activity effort, and baseline affective state. A core tenet of the model is that PAIs are most effective when activity features are matched to person characteristics (see Lyubomirsky & Layous, 2013 for a review).

The present study focused on the person–activity fit of writing a gratitude letter, examining individual changes in affect after the intervention, followed by an analysis of person features, given the limited research on individual differences in gratitude intervention outcomes (Folk & Dunn, 2023).

### 1.2. Past Research on the Effects of Gratitude PAIs Based on Person Features

#### 1.2.1. Demographics

Gratitude is associated with greater subjective well-being across age groups (Chopik et al., 2019), but findings regarding age and gratitude are mixed. Some studies report lower trait gratitude among younger and middle-aged adults compared to older adults (Chopik et al., 2019; Kern et al., 2014), while others find no associations (Kashdan et al., 2009; Wood et al., 2008b). Further, most gratitude intervention studies use undergraduate samples, limiting generalizability across age or generation (Chopik et al., 2019).

Women generally report higher gratitude than men, potentially due to gender role expectations and stereotypes, such as perceptions of male weakness and threatened masculinity (Froh & Bono, 2008; Froh et al., 2009b). For example, a set of studies found that young men viewed expressing gratitude as relatively complex and conflicting, whereas young women found it relatively interesting and exciting (Kashdan et al., 2009). In a follow-up study among older adults (*M* = 69.52), women reported feeling less burdened, less obligated, and more grateful after receiving a gift compared to men.

Research also suggests that individuals from collectivist cultures may benefit less from gratitude interventions than those from individualistic cultures (Shin et al., 2020; Shin & Lyubomirsky, 2017). In one study, U.S. and South Korean participants experienced similar well-being gains after performing acts of kindness, but Americans benefited more from gratitude interventions (Layous et al., 2013), possibly due to greater guilt and indebtedness felt by South Koreans. In a U.S.-based study, Latinx and European Americans rated gratitude as more desirable and appropriate, and reported more frequent and intense gratitude expressions than East Asian Americans (Corona et al., 2020).

Most relationship status studies have focused on romantic partners (e.g., Algoe et al., 2010; Kubacka et al., 2011). To our knowledge, no studies have reported differences in feelings of gratitude based on relationship status (e.g., single vs. in a relationship).

#### 1.2.2. Personality

Findings on personality and gratitude are mixed. Some studies have found no correlations (Breen et al., 2010), while others have identified positive associations with agreeableness, openness, conscientiousness, and extraversion, and negative associations with neuroticism (McCullough et al., 2002, 2004; Reckart et al., 2017; Szcześniak et al., 2020).

#### 1.2.3. Activity Effort

Activity effort appears to be a key predictor of PAI success (Wang et al., 2017). For example, in an 8-month longitudinal study, participants who reported exerting more effort while expressing gratitude or envisioning their best future selves experienced greater gains in life satisfaction and positive affect, relative to a control group (Lyubomirsky et al., 2011).

#### 1.2.4. Trait Gratitude

Some studies suggest that individuals lower in trait gratitude tend to benefit more from gratitude interventions possibly due to a floor effect (e.g., Froh et al., 2008; Harbaugh & Vasey, 2014; McCullough et al., 2004; Rash et al., 2011), while those higher in trait gratitude tend to benefit less potentially due to a ceiling effect (e.g., Harbaugh & Vasey, 2014). However, other studies have found that those high in trait gratitude may also experience greater benefits (Oltean et al., 2022; Toepfer et al., 2012).

#### 1.2.5. Baseline Affective States

Some evidence indicates that individuals with relatively low pre-intervention positive affect (i.e., they are less happy to begin with) show greater improvements in subjective well-being following gratitude interventions, compared to those with relatively higher levels of positive affect (Froh et al., 2009a). Like with trait gratitude, these findings may be related to floor and ceiling effects, respectively (Emmons & Mishra, 2011). However, other studies have not found such moderating effects (Rash et al., 2011).

### 1.3. The Present Study

Building on this review, the present pre-registered (https://aspredicted.org/B8D_QGJ accessed on 9 September 2025) study addressed the following research questions (RQ):

RQ_1_ Activity Fit: Do distinct clusters emerge based on changes in affect after writing a gratitude letter?
RQ_2_ Person Features: Are there individual differences (e.g., demographics, personality, pre-intervention affect) across these clusters?

#### 1.3.1. Activity Fit Predictions: Do Distinct Affect Clusters Emerge?

Based on prior literature suggesting that gratitude interventions do not uniformly improve well-being, we expected two to four affective clusters to emerge. A two-cluster solution would separate those who benefited (i.e., increased positive affect and decreased negative affect) from those who did not. Three- or four-cluster solutions might include groups showing no change or mixed emotions (i.e., increases in both positive and negative affect). These cluster labels (“benefit,” “do not benefit,” “no change,” “mixed emotions”) were used as placeholders and updated following data inspection.

#### 1.3.2. Person Feature Predictions: Who Benefits Most from Gratitude Interventions?

We predicted that the benefit cluster would include a higher proportion of women, European Americans, and Latinx individuals. If clusters reflecting no benefit or mixed emotions emerged, we expected greater representation of Asian-identifying individuals. Age, generation, and relationship status were treated as exploratory.

Given that expressing gratitude facilitates introspection and personal growth (Bono & Sender, 2018; Kashdan et al., 2009), we predicted that individuals lower in trait gratitude and baseline positive affect, and higher in baseline negative affect and neuroticism, would be more likely to belong to the benefit cluster. Other personality traits were explored without specific hypotheses.

Lastly, we predicted that individuals in the benefit cluster would report higher activity effort, given prior research on the importance of effort in PAI efficacy (Lyubomirsky & Layous, 2013).

## 2. Materials and Methods

Archival data from three gratitude studies conducted by the Positive Activities and Well-Being Lab at the University of California, Riverside, were merged and used for the present study (*N* = 487).

1. Regan, Walsh, and Lyubomirsky (Regan et al., 2022; *n* = 157), henceforth the Ways of Expressing Gratitude study (https://doi.org/10.1007/s42761-022-00160-3).
2. Walsh, Regan, Twenge, and Lyubomirsky (Walsh et al., 2022c; *n* = 231), henceforth the Optimal Way to Give Thanks study (https://doi.org/10.1007/s42761-022-00150-5).
3. Walsh, Regan, and Lyubomirsky (Walsh et al., 2022b; *n* = 99), henceforth the Thanking Parents study (https://doi.org/10.1080/17439760.2021.1991449).

The Ways of Expressing Gratitude study was conducted in 2019 with Australian adults recruited using the Pureprofile sample panel. The other two studies—Optimal Way to Give Thanks (conducted in 2020) and Thanking Parents (conducted in 2016)—included an ethnically diverse sample of undergraduate students recruited from a large public university in California. As the name suggests, Thanking Parents specifically instructed participants to write a gratitude letter to one of their parents, whereas the other two studies allowed participants to write to anyone they felt grateful towards. Ethics approval was obtained from the Institutional Review Board of the University of California, Riverside. Additional information about these studies can be found in the provided DOI links and Supplementary Materials File S1.

### 2.1. Data Preparation

To minimize confounding variables in the cluster analysis, we reviewed and compared the experimental conditions and measures across the three studies prior to merging them. We selected an experimental condition that was consistent across studies: participants wrote a gratitude letter, but did not share it. The Ways of Expressing Gratitude and the Optimal Way to Give Thanks studies included multi-day interventions. For consistency, we only used post-intervention data from the first gratitude letter to control for the effects of writing multiple letters. We also selected measures that were identical or very similar in wording across studies. After identifying common conditions and measures, we standardized variable names and item coding to ensure uniformity before merging (see S1–S9 in Supplementary Materials File S2 for an overview of the original studies and a codebook).

### 2.2. Participants

The mean age of participants in the merged dataset was 28 (*SD* = 15.7)—67% were Gen Z (born between 1995 and 2007), 12% were Millennials (born between 1980 and 1994), 9% were Gen X (born between 1965 and 1979), 10% were Baby Boomers (born between 1946 and 1964), and 2% were from the Silent Generation (born between 1925 and 1945). Over half of participants identified as female (62%). There was also a mix of relationship status, with 38% single, not in a relationship, 20% single (not defined), 19% in a relationship, 15% married, 5% divorced/separated, 3% cohabiting, and 1% widowed. The sample was ethnically diverse: 34% identified as White, 26% as Asian, 24% as Hispanic, 6% as Black, and 10% as Other. Notably, 93% of non-White participants were drawn from the undergraduate samples. A full breakdown of sample characteristics is provided in Table A1 of Appendix A.

### 2.3. Activity Fit Measures

To examine differences in the affective experience of writing a gratitude letter, we used 6 items from Schnall and colleague’s Elevation Scale (Schnall et al., 2010) and 14 items from an adapted version of the Affect Adjective Scale (AAS; Diener & Emmons, 1985). Four composite scores were created: Elevation, Positive Affect (PA), Negative Affect (NA), and Self-Conscious Affect (SCA). Change scores were calculated by subtracting pre-intervention (T_0_) ratings from post-intervention ratings (T_1_).

Elevation was measured using items such as “A warm feeling in your chest,” “Moved,” and “Uplifted,” rated on a 7-point scale ranging from 1 (*do not feel at all*) to 7 (*feel very strongly*). We computed scale reliabilities using Cronbach’s alpha (T_0_ α = 0.85; T_1_ α = 0.89).

The AAS also used a 7-point scale ranging from 1 (*not at all*) to 7 (*extremely*). PA included “Happy,” “Pleased,” “Joyful,” and “Enjoyment/Fun” (T_0_ α = 0.92; T_1_ α = 0.92). NA included “Unhappy,” “Worried/Anxious,” “Angry/Hostile,” “Frustrated,” and “Depressed/Blue” (T_0_ α = 0.87; T_1_ α = 0.86). SCA included “Indebted,” “Guilty,” “Embarrassed,” “Uncomfortable,” and “Ashamed” (T_0_ α = 0.80; T_1_ α = 0.81).

Why separate negative affect from self-conscious affect? As previously mentioned, research suggests that writing a gratitude letter may elicit both positive and self-conscious emotions (e.g., guilt, indebtedness), resulting in mixed emotional responses (Layous et al., 2017; Walsh et al., 2022a; Watkins et al., 2006). For example, a series of studies found that gratitude, but not other PAIs or control tasks, produced both pleasant and unpleasant states, including feeling moved, uplifted, and indebted (Layous et al., 2017). Another study replicated these findings, with participants reporting greater elevation, indebtedness, and guilt after writing a gratitude letter than after completing a neutral task (Walsh et al., 2022a). Therefore, a cluster may emerge in which SCA, but not NA, increases after writing a letter.

### 2.4. Person Features Measures

Following the first part of the analyses, we examined whether cluster membership varied by person features, including demographics (mean age, generation, gender, ethnicity, and relationship status), personality, trait gratitude, baseline affect, and activity effort.

Age was entered via an open-text numerical field. Generation was estimated by subtracting age from the year of the study’s pre-test (or the prior year, if the pre-test occurred in the first quarter of the year). Gender options included: “Male,” “Female,” and “Other.” Relationship status categories included: “Single, not in a relationship,” “In a relationship,” “Cohabiting/living together,” “Single (not defined),” “Married,” “Divorced/Separated,” and “Widowed.” Ethnicity items included: “Asian,” “Black,” “Hispanic,” “White,” and “Other.”

Personality was measured using the 15-item Big Five Inventory-2 Extra Short Form (BFI-2-XS; Soto & John, 2017) administered in the Ways of Expressing Gratitude and the Optimal Way to Give Thanks studies (*N* = 388). Each personality trait was assessed with three items (positively and negatively worded), rated using a 5-point Likert scale from 1 (*strongly disagree*) to 5 (*strongly agree*). Trait composite scores were calculated for extraversion (α = 0.67), agreeableness (α = 0.54), conscientiousness (α = 0.58), neuroticism (α = 0.81), and openness to experience (α = 0.44). As noted by Soto and John (2017), relatively low internal consistencies are typical for the BFI-2-XS, sometimes falling below conventional thresholds, but this is expected given that the scale’s design prioritizes content validity over internal consistency (p. 70).

Dispositional gratitude was captured using a composite score of two items from the Trait Gratitude Questionnaire (McCullough et al., 2002): “I am grateful to a wide variety of people currently in my life” and “I find myself more able to appreciate the people, events, and situations that have been part of my life history,” rated from 1 (*strongly disagree*) to 7 (*strongly agree;* α = 0.76). Baseline affect was assessed using the same T_0_ affect and elevation scales and composite scores (i.e., elevation, PA, NA, SCA) used in the cluster analyses.

Activity effort was measured using one item with a 7-point scale. Of note, question wording was less consistent across studies compared to the other measures. The Ways of expressing gratitude study asked, “During this time, how much effort did you put into your daily writing activities?” with three points in the scale labeled 1 (*Not a lot of effort*), 4 (*A moderate amount of effort*), and 7 (*A great deal of effort*). The Optimal Way to Give Thanks study asked, “How much effort did you put into writing the letter of gratitude?” with the same scale labels used in the Ways of Expressing Gratitude study. The Thanking Parents study asked, “Please select the point on the scale for each statement to indicate the extent to which you agree or disagree with the statement: I put forth my best effort in responding to this survey.” All points of the scale were labeled and ranged from 1 (*Strongly agree*) to 7 (*Strongly disagree*). Because this item was administered post-intervention across studies, it was presumed to reflect effort during the gratitude letter activity.

### 2.5. Coding the Gratitude Letters

Next, we conducted an exploratory qualitative analysis of the content of the gratitude letters. Only participants from the Ways of Expressing Gratitude and Thanking Parents studies uploaded letters (*N* = 236), and thus were included in these analyses. Four coders (three human, one AI tool—ChatGPT, version 5) were blind to study aims and received the same codebook, including definitions and scale anchors. All coders independently coded the same 20% subset of letters used to establish interrater reliability. Codebook refinements were informed by human coder discussion. Reliability estimates including and excluding the AI coder were nearly identical, indicating that inclusion of AI ratings did not meaningfully alter agreement estimates. Final coding indicated strong consistency. Intraclass correlation coefficients (ICCs) for average measures ranged from 0.85 to 0.95 (Koo & Li, 2016). Average ratings across coders were used in analyses.

Seven themes were coded on 5-point scales, informed by prior literature on gratitude letter quality (Hodge et al., 2023; Lyubomirsky & Layous, 2013; Lyubomirsky et al., 2005): (1) writer effort, (2) level of detail, (3) heartfelt sincerity, (4) reflection depth, (5) genuineness, (6) superficiality, and (7) benefactor effort (based on the reason the writer expressed gratitude). The number of words in each letter was also recorded. The finalized coding codebook, including full definitions and scale anchors for all themes, is provided in Supplementary Materials File S3.

Writer effort was coded based on the length and level of detail noted in the letter, with a scale ranging from 1 (*No effort at all*) to 5 (*A great deal of effort*). ICC(3, k) = 0.95, CI [0.94, 0.96], *F*(235, 705) = 20.45, *p* < 0.001.

Level of detail reflected the level of specificity provided when explaining the reason(s) for being grateful (e.g., specific context, anecdotes, examples), using a scale ranging from 1 (*Not at all detailed*) to 5 (*Extremely detailed*). ICC(3, k) = 0.91, CI [0.89, 0.93], *F*(235, 705) = 11.18, *p* < 0.001.

Heartfelt sincerity was coded based on the writer’s use of language expressing what it has meant or how it has felt to receive support from the benefactor, using a scale ranging from 1 (*Not heartfelt and sincere at all*) to 5 (*Extremely heartfelt and sincere*). ICC(3, k) = 0.90, CI [0.88, 0.92], *F*(235, 705) = 10.14, *p* < 0.001.

Reflection depth was based on the degree to which the writer seemed to have deeply considered the actions of the benefactor, with a scale ranging from 1 (*No depth of reflection at all*) to 5 (*A great deal of depth of reflection*). ICC(3, k) = 0.93, CI [0.91, 0.94], *F*(235, 705) = 14.23, *p* < 0.001.

Genuineness was coded based on perceptions that the letter truly expressed genuine gratitude, with a scale ranging from 1 (*Not at all genuine*) to 5 (*Extremely genuine*). ICC(3, k) = 0.89, CI [0.87, 0.91], *F*(235, 705) = 9.47, *p* < 0.001.

Superficiality rated perceptions about the letter’s substance (e.g., the degree to which the letter read like a greeting card vs. a personalized letter), with a scale ranging from 1 (*Not at all superficial*) to 5 (*Extremely superficial*). ICC(3, k) = 0.85, CI [0.81, 0.88], *F*(235, 705) = 6.56, *p* < 0.001.

Benefactor effort reflected whether the benefit conferred seemed like a heavy lift (e.g., took a considerable amount of time), based on the reason the letter writer was grateful, using a scale ranging from 1 (*No effort at all/effort not specified*) to 5 (*A great deal of effort*). The benefit conferred could be tangible (e.g., helping pay for college) or intangible (e.g., consistently providing support through a divorce or illness). ICC(3, k) = 0.86, CI [0.82, 0.88], *F*(235, 705) = 6.92, *p* < 0.001.

### 2.6. Data Exclusion Criteria

The original study exclusion criteria were followed with two exceptions. First, although the Optimal Way to Give Thanks study excluded one participant for reporting minimal effort, we retained this case due to our focus on effort. Second, participants missing pre- or post-intervention data for the cluster analysis variables (PA, NA, SCA, elevation) were excluded (*n* = 12), as change scores could not be computed.

### 2.7. Analytic Approach

Analyses were conducted using R(v4.4.2), except for column proportion comparisons of categorical variables, which were computed using SPSS(29.0.1).

Prior to conducting a person-centered cluster analysis to identify heterogeneity in affective responses to gratitude letter writing, we z-scored the change scores for PA, elevation, NA, and SCA to account for differences in ranges and standard deviations, thereby minimizing the risk of misclassification (Zakharov, 2016). We also examined the distribution and intercorrelations of the z-scored variables to reduce concerns about multicollinearity and to ensure that none of the original datasets would form its own cluster. We then addressed our first research question—do distinct clusters emerge based on changes in affect after writing a gratitude letter?—using K-means clustering with the Euclidean distance metric and centroid averaging (Jain, 2010; Zakharov, 2016).

The ideal sample size for cluster analysis depends on assumptions about the data-generating process and the stability of emergent patterns. Prior methodological work suggests that an adequate sample can be approximated by multiplying 70 by the number of variables included (Dolnicar et al., 2014); the present sample size (*N* = 487) exceeded this guideline. In addition, consistent with best practices for person-centered analytic approaches, the clustering analyses were intended to be exploratory and hypothesis-generating. Accordingly, cluster solutions are interpreted as descriptive patterns of affective response.

To identify the optimal cluster solution, we examined compactness (i.e., the homogeneity within each cluster), separability (i.e., the distinctiveness of each cluster), misclassification risk, and interpretability (Vargha et al., 2016). Compactness was assessed with a scree plot of within-cluster sum of squares (WSS). Lower WSS indicated tighter clustering (i.e., lower variability within a cluster; Vargha et al., 2016). Separability was measured using average silhouette width with values that ranged from 0 (low separability) to 1 (high separability; Rousseeuw, 1987). Misclassification risk was inspected using silhouette values for individual respondents. Numbers below zero indicated the observation may belong to another cluster. Interpretability was assessed via visualization of the clusters using principal component analyses (PCA; Pison et al., 1999) and a review of cluster characteristics based on the means of z-scored change variables. A positive z-score indicated above-average affective gains; zero indicated no change; and a negative z-score indicated a decline relative to the average change observed in the total sample.

After selecting a final cluster solution, we tested our second research question by examining whether clusters differed by person features.

One-way between-subjects ANOVAs were conducted to examine differences in mean age, baseline affect, trait gratitude, personality, activity effort, and coded features of the gratitude letters. Tukey’s Honestly Significant Difference (HSD) tests were used for post hoc comparisons (Howell, 2013). Effect sizes for the ANOVAs were estimated using eta squared (η^2^), while Cohen’s *d* was used for pairwise comparisons. Interpretations of effect size magnitude followed conventional benchmarks, with η^2^ values of 0.01, 0.06, and 0.14 considered small, medium, and large, respectively (Cohen, 1988), and Cohen’s *d* values of 0.20, 0.50, and 0.80 similarly interpreted as small, medium, and large (Cohen, 1988).

Pearson’s chi-square goodness-of-fit tests, with Benjamini–Hochberg corrections applied to adjust for multiple comparisons (Benjamini & Hochberg, 1995; Howell, 2013), were used to assess differences across clusters of categorical demographic variables. Effect sizes were estimated using adjusted Cramér’s V, with values of 0.10, 0.30, and 0.50 interpreted as small, medium, and large effects, respectively (Cohen, 1988). To reduce the risk of invalid inferences due to sparse cell sizes, categories with low counts were excluded from these analyses. This included individuals from the Silent Generation (*n* = 8), those who selected “other” as their gender identity (*n* = 2), and individuals who identified as “cohabiting” (*n* = 13) or “widowed” (*n* = 5) under relationship status.

Sensitivity power analyses were conducted using G*Power 3.1 (Faul et al., 2007) for the between-cluster comparisons. At α = 0.05 and 80% power, the study was sensitive to detect small effects for continuous outcomes analyzed with ANOVAs. Translating G*Power’s Cohen’s *f* into the η^2^ metric used in this study, the minimum detectable effect size was approximately η^2^ = 0.02 for age, baseline affect, trait gratitude, activity effort, and personality, and η^2^ = 0.04 for coded-letter variables. For categorical variables analyzed using chi-square tests, the study was similarly sensitive to detect small effects. Translating G*Power’s Cohen’s *w* into Cramer’s V, the minimum detectable effects were approximately V = 0.14 for gender, V = 0.12 for both generation and ethnicity, and V = 0.13 for relationship status. Thus, the analyses were well powered to detect small differences between clusters.

Across all analyses, an alpha level of 0.05 was used to determine statistical significance. However, significance testing was not interpreted in isolation. In line with best practices in psychological science, results were evaluated holistically, taking into account statistical significance, effect size magnitude, and the broader pattern and theoretical coherence of findings. This approach was adopted to prioritize precision in interpretation and to avoid overreliance on dichotomous thresholds alone.

## 3. Results

### 3.1. Do Distinct Affect Clusters Emerge After Writing a Gratitude Letter?

Normality was confirmed for the z-scored pre- to post-intervention changes in positive affect (PA), elevation, negative affect (NA), and self-conscious affect (SCA). Correlation analyses revealed no strong associations among the affective change variables, both overall and within each study, thereby reducing multicollinearity concerns. Additionally, the pattern of correlations was consistent across the three datasets, suggesting that no single dataset was driving cluster formation (see Table A2 and Table A3 in Appendix A).

**Figure 2:**
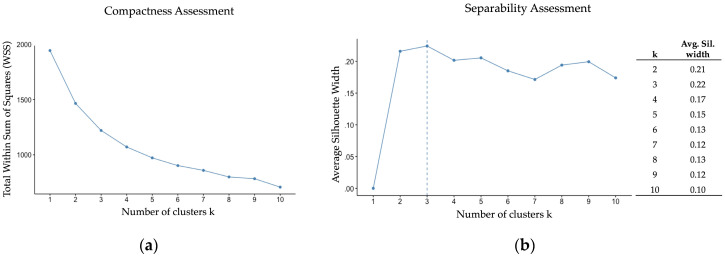
Cluster Compactness and Separability Assessment. (**a**) Scree plot indicates a two- or three-cluster solution, with a three-cluster solution providing lower WSS, indicating better compactness; (**b**) Average silhouette width indicates that a three-cluster solution provides the best separability, closely followed by a two-cluster solution.

**Figure A1:**
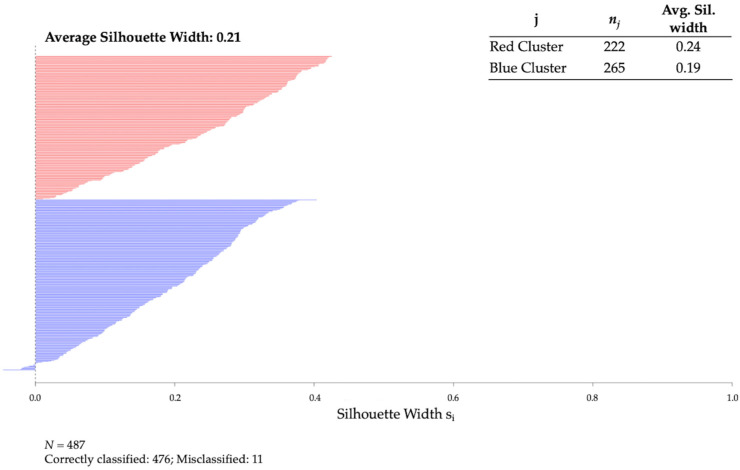
Average Silhouette Width Per Observation. Two-Cluster Solution.

**Figure A2:**
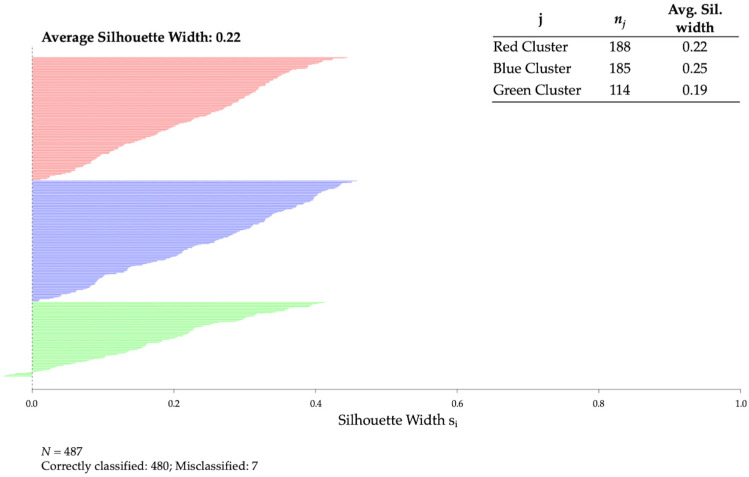
Average Silhouette Width Per Observation. Three-Cluster Solution.

The compactness analysis supported a two-cluster solution based on the elbow point criterion, but a three-cluster solution also appeared viable and had a lower within-cluster sum of squares (WSS), indicating greater compactness. The separability analysis supported the two- and three-cluster solutions (0.21 and 0.22, respectively; see Figure 2 below), relative to the other cluster solutions. Further, the silhouette plots per observation showed minimal misclassification (see Figure A1 and Figure A2 in Appendix B).

To interpret the cluster structures, we inspected the mean z-scored change scores for each solution (see Table 1). In the two-cluster solution, participants were grouped into Backfired and Uplifted clusters. The Backfired cluster was characterized by decreases in PA and elevation, increases in NA, and moderate elevations in SCA, relative to the sample average. The Uplifted cluster showed the opposite pattern, with increases in PA and elevation, decreases in NA, and moderate reductions in SCA.

In the three-cluster solution, participants were grouped in Backfired, Mixed Feelings, and Buffered clusters. Those in the Backfired cluster remained consistent with the two-cluster solution, although decreases in PA and elevation and increases in NA were more pronounced, and changes in SCA were less pronounced. Those in the Mixed Feelings cluster showed increases in PA, elevation, and SCA, but no changes in NA, relative to the sample average. Participants in the Buffered cluster experienced reductions in NA and SCA but no changes in PA or elevation.

Principal component analyses (PCAs) were conducted to visualize the cluster structure in a two-dimensional space. The first principal component (PC1) accounted for 46% of the variance and primarily reflected a contrast between positive and negative affect, with low contributions from SCA. PC1 correlated moderately with PA (*r* = 0.57), elevation (*r* = 0.52), and NA (*r* = −0.56), and weakly with SCA (*r* = −0.31). The second principal component (PC2) explained 28% of the variance and captured variation primarily driven by SCA, showing a strong positive correlation with SCA (*r* = 0.76), and weaker positive correlations with PA (*r* = 0.34), elevation (*r* = 0.44), and NA (*r* = 0.33). Full PCA loadings are shown in Table A4 of Appendix A.

**Figure 3:**
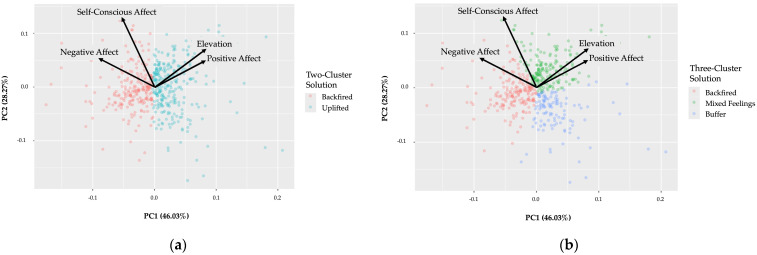
Principal Component Analyses. (**a**) Depicts observations in the two-cluster solution; (**b**) Depicts observations in the three-cluster solution.

Plots of the PCA results revealed separation between clusters. In the two-cluster solution, PC1 distinguished between positive (Uplifted) and negative (Backfired) affective responses, with minimal overlap between clusters. PC2, which captured SCA-related variation, did not clearly separate the two clusters, consistent with the absence of a distinct mixed emotion cluster. In contrast, the three-cluster solution revealed more nuanced distinctions, including a clear representation of the Mixed Feelings cluster along PC2. Although the boundaries between clusters were close, overlap remained minimal, supporting the distinctiveness of the three-cluster solution (see Figure 3).

The three-cluster solution was selected as the final model given that (1) PCA identified a mixed feelings component that accounted for a moderate amount of variance, (2) this solution allowed for a more nuanced analysis of person feature differences, and (3) this grouping had slightly better compactness and separability. Further, differences in cluster membership across the original studies were minimal (see Table A5 in Appendix A). Additional support for this choice came from examining the individual items within each affective composite (see Table A6 in Appendix A). Participants in the Buffered cluster experienced reductions in all NA and SCA items, relative to the sample average, with little to no change in PA and elevation, except for a moderate increase in happiness. The Mixed Feelings cluster showed increases in all PA, elevation, and SCA items, with the largest SCA increase observed in feelings of indebtedness (other SCA items increased moderately). The Backfired cluster exhibited decreases in all PA and elevation items and increases in all NA items. SCA also increased moderately, except for indebtedness, which showed a slight decline.

To further interpret the unexpected indebtedness change scores, post hoc correlations between indebtedness and all other affect items were examined at baseline (T_0_) and post-intervention (T_1_). Table 2 presents these associations along with significance tests. At baseline, indebtedness showed small, but statistically significant positive correlations with several elevation items (e.g., moved, uplifted), whereas post-intervention these associations strengthened to the moderate range, particularly feeling uplifted and a warm feeling in the chest, and were consistently significant. In contrast, indebtedness was moderately and significantly correlated with negative affect items at baseline; however, these associations weakened and were no longer statistically significant at T_1_. Correlations between indebtedness and other self-conscious emotions remained moderate and statistically significant at both time points, although modest reductions in magnitude were observed for feeling ashamed and uncomfortable post-intervention.

### 3.2. Who Benefits Most—And Least—From Writing a Gratitude Letter?

#### 3.2.1. Demographics

No significant differences across clusters emerged for age, *F*(2, 484) = 0.48, *p* = 0.620, η^2^ = 0.002. Similarly, generational differences across clusters were not statistically significant overall, χ^2^(6) = 11.45, *p* = 0.075, Adjusted Cramer’s V = 0.08. However, post hoc comparisons indicated that participants in the Backfired and Mixed Feelings clusters were more likely to be members of Gen Z (69% and 70%, respectively), compared to the Buffered cluster (58%, *p* < 0.05). No statistically significant differences emerged for gender (χ^2^(2) = 1.13, *p* = 0.57, Adjusted Cramer’s V < 0.001) or ethnicity (χ^2^(8) = 8.49, *p* = 0.39, Adjusted Cramer’s V = 0.02), though descriptive trends aligned with prior research. Women were slightly more likely to fall into the Buffered cluster (66%) compared to the Backfired cluster (60%), while men were more likely to appear in the Backfired cluster (40%) than the Buffered cluster (33%). Asian participants were more likely to fall into the Backfired and Mixed Feelings clusters (27% and 30%, respectively) than into the Buffered cluster (18%). White participants were more prevalent in the Buffered cluster (41%) compared to the Mixed Feelings and Backfired clusters (32% each). No differences were observed by relationship status (χ^2^(8) = 6.38, *p* = 0.60, Adjusted Cramer’s V < 0.001). Demographics per cluster are shown in Table A7 of Appendix A.

#### 3.2.2. Personality

Significant personality differences emerged across clusters. Neuroticism varied by cluster with a medium effect size, *F*(2, 385) = 13.63, *p* < 0.001, η^2^ = 0.07. Participants in the Buffered cluster scored significantly higher in neuroticism (*M* = 3.67, *SD* = 1.00) than those in the Backfired (*M* = 3.06, *SD* = 1.11) and Mixed Feelings (*M* = 2.96, *SD* = 1.07) clusters. Agreeableness also differed significantly across clusters, *F*(2, 385) = 4.66, *p* = 0.01, η^2^ = 0.02, with participants in the Mixed Feelings cluster scoring higher (*M* = 3.88, *SD* = 0.78) than those in the Buffered cluster (*M* = 3.57, *SD* = 0.77). Marginally significant effects were found for conscientiousness, *F*(2, 385) = 3.17, *p* = 0.043, η^2^ = 0.02, and openness, *F*(2, 385) = 2.87, *p* = 0.058, η^2^ = 0.01. Those in the Buffered cluster were less conscientious (*M* = 3.23, *SD* = 0.85) and less open (*M* = 3.34, *SD* = 0.85) than those in the Backfired cluster (*M* = 3.51, *SD* = 0.87; *M* = 3.59, *SD* = 0.73, respectively). No significant differences were found for extraversion (*F*(2, 385) = 2.13, *p* = 0.12, η^2^ = 0.01). Descriptive statistics and pairwise comparisons are summarized in Table A8 and Table A9.

#### 3.2.3. Baseline Affect and Trait Gratitude

Baseline affect and trait gratitude also varied significantly by cluster. Large effect sizes were observed for baseline negative affect, *F*(2, 484) = 81.25, *p* < 0.001, η^2^ = 0.25, and self-conscious affect, *F*(2, 484) = 70.27, *p* < 0.001, η^2^ = 0.23. Participants in the Buffered cluster began with higher levels of negative affect (*M* = 3.85, *SD* = 1.30) and self-conscious affect (*M* = 2.80, *SD* = 1.10) compared to both the Backfired (*M* = 2.29, *SD* = 1.04; *M* = 1.71, *SD* = 0.82, respectively) and Mixed Feelings (*M* = 2.35, *SD* = 1.07; *M* = 1.61, *SD* = 0.85, respectively) clusters. Statistically significant, though smaller, differences also emerged for baseline positive affect (*F*(2, 484) = 14.03, *p* < 0.001, η^2^ = 0.05), elevation (*F*(2, 484) = 6.87, *p* = 0.001, η^2^ = 0.03), and trait gratitude (*F*(2, 484) = 7.09, *p* < 0.001, η^2^ = 0.03). Participants in the Backfired cluster had the highest baseline positive affect (*M* = 4.47, *SD* = 1.31) and elevation (*M* = 4.18, *SD* = 1.24), followed by the Mixed Feelings (*M* = 3.95, *SD* = 1.29; *M* = 3.77, *SD* = 1.20, respectively) and Buffered (*M* = 3.70, *SD* = 1.37; *M* = 3.73, *SD* = 1.34, respectively) clusters. Trait gratitude was highest among participants in the Backfired (*M* = 5.89, *SD* = 1.01) and Mixed Feelings (*M* = 5.73, *SD* = 1.12) clusters compared to the Buffered cluster (*M* = 5.39, *SD* = 1.31). Full results are presented in Table A10 and Table A11 of Appendix A.

#### 3.2.4. Activity Effort

Activity effort varied modestly across clusters, *F*(2, 434) = 3.10, *p* = 0.046, η^2^ = 0.01. Although post hoc pairwise comparisons were not statistically significant, participants in the Mixed Feelings (*M* = 5.71, *SD* = 1.09) and Buffered (*M* = 5.65, *SD* = 1.24) clusters reported slightly higher effort than those in the Backfired cluster (*M* = 5.40, *SD* = 1.21). See Table A10 and Table A11 in Appendix A for full comparisons.

#### 3.2.5. Coded Gratitude Letters

Analyses of the content of the gratitude letters revealed statistically significant differences across clusters for all seven coded categories, including writer effort (*F*(2, 233) = 4.49, *p* = 0.012, η^2^ = 0.04); level of detail (*F*(2, 233) = 6.29, *p* = 0.002, η^2^ = 0.05); heartfelt sincerity (*F*(2, 233) = 3.74, *p* = 0.025, η^2^ = 0.03); reflection depth (*F*(2, 233) = 5.28, *p* = 0.006, η^2^ = 0.04); genuineness (*F*(2, 233) = 5.12, *p* = 0.007, η^2^ = 0.04); superficiality (*F*(2, 233) = 5.64, *p* = 0.004, η^2^ = 0.05); and benefactor effort (*F*(2, 233) = 4.59, *p* = 0.011, η^2^ = 0.04). Letters from participants in the Mixed Feelings cluster were rated significantly higher on writer effort, level of detail, reflection depth, and genuineness than those from both the Backfired and Buffered clusters. Letters from those in the Mixed Feelings cluster also received higher ratings for heartfelt sincerity compared to those from the Backfired cluster and included more references to benefactor effort compared to the Buffered cluster. Letters from the Backfired cluster received higher superficiality ratings compared to those from the Mixed Feelings cluster. Finally, letter word count varied marginally across clusters, *F*(2, 233) = 2.99, *p* = 0.052, η^2^ = 0.03. Participants in the Mixed Feelings cluster (*M* = 135.56, *SD* = 75.04) wrote slightly longer letters than those in the Buffered cluster (*M* = 107.05, *SD* = 68.67). Mean ratings and standard deviations for each cluster are presented in Table 3, with pairwise comparisons detailed in Table A12 of Appendix A.

## 4. Discussion

The primary aim of this study was to evaluate individual differences in the person–activity fit (Lyubomirsky & Layous, 2013) of writing a gratitude letter by examining heterogeneity in emotional responses to this intervention and exploring who benefits most and least. To this end, we merged data from three existing experiments in which participants wrote gratitude letters but did not share them. These analyses address limitations of past research focused on mean-level intervention effects, which mask distinct patterns of affective change across individuals.

### 4.1. Do Distinct Affect Clusters Emerge After Writing a Gratitude Letter?

Both two- and three-cluster solutions emerged from the analysis with comparable compactness and separability. However, the three-cluster solution enhanced interpretability, allowed for more nuanced analyses, and aligned closely with prior work on gratitude-induced mixed affective states (e.g., Layous et al., 2017).

Findings partially supported our predictions. The Backfired and Mixed Feelings clusters emerged as expected, but a distinct Benefit cluster did not. Instead, the Buffered cluster comprised individuals who, on average, experienced decreases in negative affect and self-conscious affect. The Mixed Feelings cluster included individuals who showed increases in positive affect, elevation, and self-conscious affect. In contrast, individuals in the Backfired cluster experienced decreases in positive affect and elevation and increases in negative affect.

An exploratory follow-up revealed that, among individuals in the Mixed Feelings cluster, indebtedness increased the most relative to other self-conscious affect items. In the Backfired cluster, indebtedness slightly decreased while all other self-conscious affect items moderately increased. Further, across the full sample, the meaning of indebtedness appeared to shift after the intervention. Prior to the intervention, indebtedness was modestly associated with both elevation and negative affect, suggesting an affectively mixed or ambiguous emotional profile. Following gratitude letter writing, indebtedness became more strongly and selectively aligned with elevation-related emotions—such as feeling moved, uplifted, and motivated toward prosocial behavior—while its associations with negative affect diminished to near zero. Notably, indebtedness remained robustly correlated with other self-conscious emotions at both time points, indicating that the intervention altered its affective embedding rather than its self-evaluative character. This shift suggests that the Mixed Feelings cluster may not reflect emotional ambivalence in the traditional sense of simultaneous positive and negative affect, but instead may represent a distinct pattern in which self-conscious emotions are embedded within an overall uplifted emotional profile—consistent with prior findings that gratitude interventions uniquely elicit both uplifting and self-evaluative emotions (Layous et al., 2017; Watkins et al., 2006).

These findings underscore the dual nature of gratitude. Self-conscious emotions such as indebtedness, when felt in a relational context, can reinforce prosocial norms and deepen social bonds (Hareli & Parkinson, 2008; Tsang, 2006, 2007). Of note, the present study measured indebtedness using a single adjective item without an explicit referent, which may have been ambiguous prior to the intervention. However, the post-intervention shift in its correlational pattern, away from negative affect and toward elevation, suggests that the gratitude letter provided a salient interpersonal frame that transformed the meaning of indebtedness. This pattern is consistent with prior work positioning indebtedness as a relational emotion central to prepositional gratitude (i.e., grateful towards someone; Nelson et al., 2024) rather than a uniformly aversive self-conscious state. Past research has also distinguished between transactional (need to repay) and transcendent (desire to pay) indebtedness, with the latter predicting greater prosocial reciprocity (Nelson et al., 2023). Future research may benefit from isolating this measure rather than combining it with other self-conscious emotions, and from using additional validated measures, to replicate these findings and to further examine the unique psychological and relational consequences of indebtedness.

### 4.2. Who Benefits Most—And Least—From Writing a Gratitude Letter?

No significant differences in emotional response clusters emerged based on demographic features such as gender, ethnicity, age or generation. This finding suggests that demographics alone may be insufficient to predict who will benefit from gratitude letter writing.

In contrast, baseline emotional states, personality traits, and features of the gratitude letters were more informative. Individuals in the Buffered cluster—those who experienced reductions in negative affect and self-conscious affect—had higher pre-intervention levels of neuroticism, negative affect, and self-conscious affect, along with lower levels of trait gratitude. Effect sizes were small for trait gratitude, medium for neuroticism, and large for negative affect and self-conscious affect. These findings align with prior work showing that gratitude interventions are especially beneficial for individuals who begin with low trait gratitude or higher negative emotionality, likely due to a floor effect (Froh et al., 2008, 2009a; Harbaugh & Vasey, 2014; McCullough et al., 2004).

Individuals in the Mixed Feelings cluster were more grateful and seemed more agreeable than individuals in the Buffered cluster. Further, according to coder ratings, those in the Mixed Feelings cluster invested more effort, with letters that were more detailed, reflective, and genuine relative to the other clusters, and more heartfelt and sincere compared to the Backfired cluster. Further, coders rated the benefactor’s actions described in these letters as more effortful compared to those described by individuals in the Buffered cluster. Although effect sizes were small, findings suggest that individuals who experience both uplifting and self-conscious emotional responses may be tapping into the social–relational meaning of gratitude. Prior studies support the view that mixed emotions—particularly those that include a sense of indebtedness—can promote social connection and motivate reciprocity (Watkins et al., 2006; Nelson et al., 2023).

In contrast, individuals in the Backfired cluster began with relatively high levels of positive affect, elevation, and trait gratitude, and seemed to have higher levels of openness and conscientiousness. Following the intervention, these individuals experienced greater increases in feelings such as frustration and unhappiness. Further, coders rated their letters as more superficial compared to letters from the Mixed Feelings cluster. Earlier work suggests that when individuals are already dispositionally grateful and in a positive mood, gratitude interventions may yield little benefit due to a ceiling effect (Emmons & Mishra, 2011; Harbaugh & Vasey, 2014). However, although effect sizes are small, the present findings may be the first to suggest that writing a gratitude letter can backfire among individuals with certain emotional or dispositional profiles. Writing a gratitude letter may have disrupted a previously positive state, or the activity may have felt contrived or inauthentic, producing frustration or disengagement.

These findings also align with recent research on tailoring interventions based on emotional readiness. A recent study of Chinese adults found that an AI-powered system that delivered interventions matched to participants’ baseline affect produced the strongest gains in well-being relative to other experimental conditions (Liu et al., 2024). Specifically, individuals high in positive affect were encouraged to reflect on past accomplishments, while those low in positive affect engaged in best-self visualizations, and the remaining participants engaged in a gratitude activity. Combined with the present study, these findings suggest that tailoring activities to person–state features may enhance psychological outcomes. Future research should replicate this work.

### 4.3. Limitations

Merging datasets presented both strengths and limitations for this study. A primary strength was the increased sample size and diversity, which allowed for more stable cluster estimation and detection of meaningful individual differences in affective response. Larger and more heterogeneous samples help address the overreliance on convenience samples (e.g., college students) often seen in gratitude intervention research (e.g., Chopik et al., 2019; Froh et al., 2009b). At the same time, despite a thorough review to ensure consistency across studies, some differences remained (see Section 2).

Although the gratitude letter activity was highly similar across studies, methodological differences beyond our core variables may have been introduced. To help maintain consistency, we only included measures with similar wording across studies and used z-scored change scores to limit the influence of variable scale differences and unequal variances. Nevertheless, unmeasured methodological differences cannot be ruled out. Given that this approach limited variable inclusion, future studies could incorporate other affective and cognitive states (e.g., authenticity, social connection, rumination) and person features (e.g., motivation, self-efficacy, socioeconomic status) to further inform individual differences across clusters.

Further, although we found minimal differences in cluster membership by study, the merged datasets differed in participant age, cultural context, and letter targets (e.g., parents vs. freely chosen recipients). As such, some demographic patterns may partially reflect study-specific recruitment or protocol features. Cultural and generational differences in emotional expression, particularly for self-conscious emotions, may also influence affective reporting, even though age, generation, and ethnicity did not statistically differentiate clusters in the present analyses.

Personality traits were assessed using the BFI-2-XS, which prioritizes content coverage over internal consistency (Soto & John, 2017). Accordingly, reliability was modest for extraversion, agreeableness, conscientiousness, and openness, and findings involving these traits should be interpreted as exploratory. Future work could examine whether more reliable personality assessments can meaningfully inform intervention tailoring. 

In addition, effect sizes in the person feature comparisons were small, with the exception of baseline negative affect, self-conscious affect, and neuroticism. This pattern aligns with meta-analyses of gratitude interventions, which have generally reported small-to-medium effects (Dickens, 2017). In psychological science, small effects are common due to the complex nature of human behavior (Götz et al., 2021). Importantly, small but statistically significant effects can still offer valuable theoretical and applied insights, especially when considered in context (Funder & Ozer, 2019). This study contributes to that goal by highlighting patterns that may otherwise be obscured in group-level averages.

Although cluster sizes were sufficient for the primary analyses, the study was not designed to test higher-order interactions or fine-grained subgroup effects. Accordingly, findings should be interpreted as descriptive of observed patterns rather than definitive tests of moderation. Moreover, the analytic sample was not demographically representative. Women, Gen Z participants, and single individuals were overrepresented. Further, due to chi-square test sensitivity to small expected frequencies, we excluded underrepresented categories from the demographic person feature analyses, including participants from the Silent Generation, individuals who selected “other” gender identities, and those reporting “cohabiting” or “widowed” relationship statuses. Future studies should aim to replicate these findings using more demographically balanced and population-representative samples.

This study also focused exclusively on nonclinical samples. While the present findings suggest that individuals higher in negative and self-conscious affect may experience the greatest reductions in these feelings after a gratitude letter intervention, prior research raises important caveats. For instance, individuals with clinical symptoms of depression or anxiety who are prone to experiencing guilt, shame, or embarrassment may not benefit from gratitude interventions (Layous et al., 2017). Therefore, clinical applications of gratitude interventions must be guided by careful assessment and tailoring. Future studies could examine clustering patterns in clinical populations to explore whether similar or distinct emotional profiles emerge.

In addition, the findings pertain specifically to writing a gratitude letter without sharing it. These results should not be generalized to other forms of gratitude practice, such as counting blessings or delivering the letter to the intended recipient. Prior research suggests that delivering a gratitude letter can produce stronger or different emotional effects than writing alone (Kumar & Epley, 2018; Walsh et al., 2022c). Future studies should extend this clustering approach to a broader range of gratitude and positive activity interventions (e.g., acts of kindness, best-self visualizations, practicing optimism) to examine whether similar person–activity fit patterns emerge.

Finally, although the present analyses were preregistered, the person-centered clustering approach is inherently exploratory, and the identified affective profiles should be replicated using independent samples.

## 5. Conclusions

Writing a gratitude letter may not be universally beneficial. The present findings highlight the importance of considering differences in individual moods and traits prior to using gratitude letter interventions to improve well-being.

*Who might benefit?*

Writing a gratitude letter may boost positive feelings along with feelings of indebtedness among individuals who put effort into the activity (e.g., write letters that are detailed and genuine), and even among some who may already have the tools to be dispositionally grateful. Notably, feeling indebted may not necessarily be negative for some individuals and, based on prior research, may promote reciprocity.

Writing a gratitude letter may also shield certain individuals against negative emotions—specifically, those with room to improve their moods, those who tend to be less grateful, and those who are dispositionally neurotic (e.g., worries a lot, is easily upset). The introspective feature of expressing gratitude may help this profile of individual foster a sense of coherence—the perception that the world is comprehensible, manageable, and meaningful (Antonovsky, 1993; Lindström & Eriksson, 2005)—and motivate positive reframing of unfavorable events into manageable ones (Lambert et al., 2009), thus promoting personal growth.

*Who might not benefit as much?*

Individuals who are already in a good mood and some who are dispositionally grateful may experience ceiling or backfiring effects. In other words, rather than providing an incremental mood boost, this activity may lead to the opposite effect among those who are relatively happier and grateful.

## Figures and Tables

**Table 1:** Mean Affect z-Scored Change Score.

	Two-Cluster Solution		Three-Cluster Solution		
Composite Score	Backfired	Uplifted	Backfired	Mixed Feelings	Buffered
Positive Affect	−0.60	0.51	−0.69	0.64	0.07
Elevation	−0.58	0.48	−0.72	0.68	0.06
Negative Affect	0.61	−0.51	0.66	−0.03	−1.02
Self-Conscious Affect	0.32	−0.27	0.20	0.45	−1.06
Total (*n*)	222	265	185	188	114

Note. Total *N* = 487. Values represent z-scored affect change scores. Positive values indicate increases from pre- to post-intervention; negative values indicate decreases.

**Table 2:** Correlation of Indebtedness with Other Affect Items.

		*r* Indebtedness	
Composite Score	Items	T_0_	T_1_
Positive Affect	Happy	−0.01	0.05
	Pleased	0.06	0.14 ***
	Joyful	0.09	0.07
	Enjoyment/Fun	0.09 *	0.06
Elevation	Moved	0.20 ***	0.29 ***
	Uplifted	0.09	0.24 ***
	Optimistic about humanity	0.13 ***	0.21 ***
	A warm feeling in your chest	0.14 ***	0.27 ***
	A desire to help others	0.12 *	0.19 ***
	A desire to become a better person	0.06	0.12 *
Negative Affect	Unhappy	0.23 ***	0.07
	Worried/Anxious	0.14 ***	−0.06
	Angry/Hostile	0.25 ***	0.08
	Frustrated	0.26 ***	0.01
	Depressed/Blue	0.13 ***	0.05
Self-Conscious Affect	Indebted	1	1
	Guilty	0.27 ***	0.28 ***
	Embarrassed	0.32 ***	0.28 ***
	Uncomfortable	0.21 ***	0.15 ***
	Ashamed	0.38 ***	0.29 ***

Note. *N* = 487. Values are Pearson correlations between indebtedness and affect items at baseline (T_0_) and post-intervention (T_1_). Correlations were examined to assess changes in the affective embedding of indebtedness following gratitude letter writing. * *p* < 0.05, *** *p* < 0.001.

**Table 3:** Gratitude Letter Coding Theme and Word Count—Means and Standard Deviations Per Cluster.

	Backfired		Mixed Feelings		Buffered	
	(A)		(B)		(C)	
Coding Theme	*Mean*	*SD*	*Mean*	*SD*	*Mean*	*SD*
Writer effort	2.99	1.31	3.43 ^AC^	1.06	2.95	1.10
Level of detail	2.63	1.18	3.13 ^AC^	1.00	2.62	1.06
Heartfelt sincerity	3.14	1.20	3.53 ^A^	0.88	3.18	0.97
Reflection depth	2.69	1.22	3.17 ^AC^	1.07	2.68	1.12
Genuineness	3.10	1.25	3.54 ^AC^	0.94	3.07	1.02
Superficiality	2.34 ^B^	1.17	1.84	0.80	2.16	0.99
Benefactor Effort	2.68	1.15	2.98 ^C^	0.98	2.48	0.97
Word Count	114.61	86.94	135.56	75.04	107.05	68.67

Note. Total *N* = 236 (Backfired *n* = 76, Mixed Feelings *n* = 96, Buffered *n* = 64). Significance level (*p* < 0.05) denoted with superscripts of upper case letters (A, B, C): For each significant pair of means, the letter of the category with the smaller column proportion appears in the category with the larger column proportion. For example, letters from participants in the Mixed Feelings cluster were rated significantly higher on writer effort than those from both the Backfired and Buffered clusters.

## Data Availability

The raw data supporting the conclusions of this article will be made available by the authors on request.

## Supplementary Materials

The following supporting information can be downloaded at: https://www.mdpi.com/article/10.3390/bs16020232/s1.

## Appendix A. Tables

**Table A1:** Demographics.

Baseline Characteristic	Present Study	Ways of Expressing Gratitude Study	Optimal Way to Give Thanks Study	Thanking Parents Study
Age (*M* ± *SD* in years)	28 (15.7)	47 (15.6)	19 (1.85)	19 (1.5)
Generation				
Gen Z	67%	5%	99%	91%
Millennial	12%	30%	1%	9%
Gen X	9%	28%	0%	0%
Boomer	10%	32%	0%	0%
Silent	2%	5%	0%	0%
Sex				
Female	62%	50%	66%	73%
Male	38%	50%	33%	27%
Other	0%	0%	1%	NA
Ethnicity				
White	34%	85%	8%	14%
Black	6%	10%	3%	9%
Hispanic	24%	0%	35%	35%
Asian	26%	0%	44%	24%
Other	10%	5%	10%	17%
Relationship Status				
Single, not in a relationship	38%	17%	69%	NA
Relationship	19%	15%	29%	NA
Cohabiting	3%	8%	0%	NA
Single (not defined)	20%	NA	NA	100%
Married	15%	44%	1%	0%
Divorced/Separated	5%	14%	0%	0%
Widowed	1%	3%	0%	0%
Total	487	157	231	99

Note. Sample characteristics are shown for the total sample and for each of the individual studies that were merged to create the present dataset. Values represent column percentages unless otherwise noted. Percentages may not sum to 100 due to rounding. NA = Not asked.

**Table A2:** Correlation of z-Scored Change Score Variables—Present Study.

	PA	Elev	NA	SCA
Positive Affect (PA)	1			
Elevation (Elev)	0.48 ***	1		
Negative Affect (NA)	−0.37 ***	−0.27 ***	1	
Self-Conscious Affect (SCA)	−0.06	−0.02	0.39 ***	1

Note. Values are Pearson correlations among z-scored affect change scores (post-intervention minus baseline) for the full sample. Correlations were examined to assess associations among affective change variables and to evaluate potential multicollinearity prior to cluster analysis. Overall, correlations were modest in magnitude, indicating limited multicollinearity. *** *p* < 0.001.

**Table A3:** Correlation of z-Scored Change Score Variables—Datasets Used in the Present Study.

	Ways of Expressing Gratitude Study				Optimal Way to Give Thanks Study				Thanking Parents Study			
	PA	Elev	NA	SCA	PA	Elev	NA	SCA	PA	Elev	NA	SCA
Positive Affect (PA)	1				1				1			
Elevation (Elev)	0.46 ***	1			0.56 ***	1			0.38 ***	1		
Negative Affect (NA)	−0.34 ***	−0.10	1		−0.51 ***	−0.48 ***	1		−0.32 ***	0.01	1	
Self-Conscious Affect (SCA)	−0.01	−0.01	0.41 ***	1	−0.07	−0.13	0.39 ***	1	−0.15	0.15	0.38 ***	1

Note. Values are Pearson correlations among z-scored affect change scores (post-intervention minus baseline), computed separately for each of the three datasets that were merged to create the present sample. Correlations were examined to evaluate potential multicollinearity and to assess whether the pattern of associations among affective change variables was consistent across studies. Across all three datasets, correlations were modest and followed a similar pattern, suggesting that no single dataset disproportionately influenced cluster formation. *** *p* < 0.001.

**Table A4:** Principal Component Analysis (z-Scored Change Scores).

	PC1	PC2
Positive Affect	0.57	0.34
Elevation	0.52	0.44
Negative Affect	−0.56	0.33
Self-Conscious Affect	−0.31	0.76
Standard Deviation	1.36	1.06
Proportion of Variance	0.46	0.28
Cumulative Proportion	0.46	0.74

Note. PC = Principal Component. PC1 primarily reflects a contrast between positive and negative affect, whereas PC2 captures variation driven primarily by self-conscious affect. Full loadings are shown in the table.

**Table A5:** Cluster Membership.

	Present Study	Ways of Expressing Gratitude Study	Optimal Way to Give Thanks Study	Thanking Parents Study
		(A)	(B)	(C)
Backfired	38%	33%	42%	35%
Mixed Feelings	39%	34%	40%	43%
Buffered	23%	33% ^B^	18%	21%
Total	487	157	231	99

Note. Values represent column percentages for the total sample and for each of the individual studies that were merged to create the present dataset. Significance level (*p* < 0.05) denoted with superscripts of upper case letters (A, B, C): For each significant pair of means, the letter of the category with the smaller column proportion appears in the category with the larger column proportion.

**Table A6:** Mean Affect z-Scored Change Score Per Item for the Three-Cluster Solution.

Composite Score	Items	Backfired	Mixed Feelings	Buffered
Positive Affect	Happy	−0.63	0.47	0.25
	Pleased	−0.58	0.51	0.10
	Joyful	−0.58	0.54	0.05
	Enjoyment/Fun	−0.45	0.52	−0.13
Elevation	Moved	−0.51	0.52	−0.04
	Uplifted	−0.63	0.55	0.11
	Optimistic about humanity	−0.46	0.46	−0.01
	A warm feeling in your chest	−0.57	0.50	0.11
	A desire to help others	−0.44	0.39	0.06
	A desire to become a better person	−0.38	0.39	−0.01
Negative Affect	Unhappy	0.54	−0.02	−0.83
	Worried/Anxious	0.40	−0.10	−0.49
	Angry/Hostile	0.42	0.11	−0.88
	Frustrated	0.53	−0.06	−0.76
	Depressed/Blue	0.48	−0.03	−0.74
Self-Conscious Affect	Indebted	−0.16	0.46	−0.51
	Guilty	0.18	0.22	−0.66
	Embarrassed	0.20	0.25	−0.73
	Uncomfortable	0.29	0.18	−0.76
	Ashamed	0.21	0.27	−0.78
Total (*n*)		185	188	114

Note. Total *N* = 487. Values represent z-scored affect change scores. Positive values indicate increases from pre- to post-intervention; negative values indicate decreases.

**Table A7:** Demographics Per Cluster.

Baseline Characteristic	Backfired	Mixed Feelings	Buffered
	(A)	(B)	(C)
Age (*M* ± *SD* in years)	27 (15.0)	28 (17.2)	29 (14.2)
Generation			
Gen Z	69% ^C^	70% ^C^	58%
Millennial	12%	9%	18%
Gen X	9%	6%	14%
Boomer	9%	12%	11%
Silent	2%	3%	0%
Sex			
Female	60%	62%	66%
Male	40%	38%	33%
Other	1%	0%	1%
Ethnicity			
White	32%	32%	41%
Black	7%	5%	9%
Hispanic	25%	23%	24%
Asian	27%	30%	18%
Other	10%	10%	9%
Relationship Status			
Single, no relationship	41%	36%	37%
Relationship	20%	19%	17%
Cohabiting	3%	3%	2%
Single (not defined)	19%	23%	18%
Married	14%	13%	18%
Divorced/Separated	4%	4%	8%
Widowed	1%	2%	0%
Total (*n*)	185	188	114

Note. *N* = 487. Sample characteristics are shown for each cluster. Values represent column percentages unless otherwise noted. Percentages may not sum to 100 due to rounding. Significance level (*p* < 0.05) denoted with superscripts of upper case letters (A, B, C): For each significant pair of means, the letter of the category with the smaller column proportion appears in the category with the larger column proportion.

**Table A8:** Personality—Means and Standard Deviations Per Cluster.

	Backfired		Mixed Feelings		Buffered	
	(A)		(B)		(C)	
Personality	*Mean*	*SD*	*Mean*	*SD*	*Mean*	*SD*
Neuroticism	3.06	1.11	2.96	1.07	3.67 ^AB^	1.00
Agreeableness	3.73	0.73	3.88 ^C^	0.78	3.57	0.77
Conscientiousness	3.51 ^C^	0.87	3.45	0.89	3.23	0.85
Openness	3.59 ^C^	0.73	3.47	0.78	3.34	0.85
Extraversion	3.02	0.95	2.82	0.93	2.81	0.93

Note. Total *N* = 388 (Backfired *n* = 150, Mixed Feelings *n* = 145, Buffered *n* = 93). Significance level (*p* < 0.05) denoted with superscripts of upper case letters (A, B, C): For each significant pair of means, the letter of the category with the smaller column proportion appears in the category with the larger column proportion.

**Table A9:** Personality—Cluster Pairwise Comparison Statistics.

Personality Type	Contrast	Diff	*p* Adj	Cohen’s *d*
Neuroticism	Mixed Feelings-Backfired	−0.10	0.718	−0.09
Neuroticism	Buffered-Backfired	0.61	<0.000 ***	0.57
Neuroticism	Buffered-Mixed Feelings	0.71	<0.000 ***	0.66
Agreeableness	Mixed Feelings-Backfired	0.14	0.241	0.19
Agreeableness	Buffered-Backfired	−0.16	0.233	−0.22
Agreeableness	Buffered-Mixed Feelings	−0.31	0.007 *	−0.40
Conscientiousness	Mixed Feelings-Backfired	−0.07	0.797	−0.07
Conscientiousness	Buffered-Backfired	−0.28	0.037 *	−0.33
Conscientiousness	Buffered-Mixed Feelings	−0.22	0.142	−0.25
Openness	Mixed Feelings-Backfired	−0.12	0.386	−0.15
Openness	Buffered-Backfired	−0.24	0.047	−0.31
Openness	Buffered-Mixed Feelings	−0.12	0.452	−0.16
Extraversion	Mixed Feelings-Backfired	−0.19	0.177	−0.21
Extraversion	Buffered-Backfired	−0.21	0.204	−0.23
Extraversion	Buffered-Mixed Feelings	−0.02	0.990	−0.02

Note. Total *N* = 388 (Backfired *n* = 150, Mixed Feelings *n* = 145, Buffered *n* = 93). * *p* < 0.05, *** *p* < 0.001. Pairwise comparisons were conducted following significant omnibus one-way ANOVAs. Reported *p* values are Tukey-adjusted for multiple comparisons. Cohen’s *d* reflects standardized mean differences between clusters. Positive difference scores indicate higher values for the first-listed cluster.

**Table A10:** Baseline Affect, Trait Gratitude and Activity Effort—Means and Standard Deviations Per Cluster.

	Backfired		Mixed Feelings		Buffered	
	(A)		(B)		(C)	
Personality	*Mean*	*SD*	*Mean*	*SD*	*Mean*	*SD*
Positive Affect	4.47 ^BC^	1.31	3.95	1.29	3.70	1.37
Elevation	4.18 ^BC^	1.24	3.77	1.20	3.73	1.34
Negative Affect	2.29	1.04	2.35	1.07	3.85 ^AB^	1.30
Self-Conscious Affect	1.71	0.82	1.61	0.85	2.80 ^AB^	1.10
Trait Gratitude	5.89 ^C^	1.01	5.73 ^C^	1.12	5.39	1.31
Activity Effort	5.40	1.21	5.71	1.09	5.65	1.24

Note. Total Affect and Trait Gratitude *N* = 487 (Backfired *n* = 185; Mixed Feelings *n* = 188; Buffered *n* = 114). Activity Effort *N* = 437 (Backfired *n* = 163; Mixed Feelings *n* = 179; Buffered *n* = 95). Significance level (*p* < 0.05) denoted with superscripts of upper case letters (A, B, C): For each significant pair of means, the letter of the category with the smaller column proportion appears in the category with the larger column proportion.

**Table A11:** Baseline Affect, Trait Gratitude and Activity Effort—Cluster Pairwise Comparison Statistics.

Measure	Contrast	Diff	*p* Adj	Cohen’s *d*
Positive Affect T0	Mixed Feelings-Backfired	−0.52	<0.000 ***	−0.40
Positive Affect T0	Buffered-Backfired	−0.78	<0.000 ***	−0.59
Positive Affect T0	Buffered-Mixed Feelings	−0.25	0.243	−0.19
Elevation T0	Mixed Feelings-Backfired	−0.42	0.004 **	−0.33
Elevation T0	Buffered-Backfired	−0.45	0.007 **	−0.36
Elevation T0	Buffered-Mixed Feelings	−0.04	0.967	−0.03
Negative Affect T0	Mixed Feelings-Backfired	0.06	0.857	0.05
Negative Affect T0	Buffered-Backfired	1.55	<0.000 ***	1.39
Negative Affect T0	Buffered-Mixed Feelings	1.49	<0.000 ***	1.34
Self-Conscious Affect T0	Mixed Feelings-Backfired	−0.10	0.535	−0.11
Self-Conscious Affect T1	Buffered-Backfired	1.09	<0.000 ***	1.21
Self-Conscious Affect T2	Buffered-Mixed Feelings	1.19	<0.000 ***	1.32
Trait Gratitude	Mixed Feelings-Backfired	−0.17	0.331	−0.15
Trait Gratitude	Buffered-Backfired	−0.50	0.001 **	−0.45
Trait Gratitude	Buffered-Mixed Feelings	−0.34	0.032	−0.30
Effort	Mixed Feelings-Backfired	0.30	0.050	0.26
Effort	Buffered-Backfired	0.25	0.230	0.21
Effort	Buffered-Mixed Feelings	−0.06	0.922	−0.05

Note. Total Affect and Trait Gratitude *N* = 487 (Backfired *n* = 185; Mixed Feelings *n* = 188; Buffered *n* = 114). Activity Effort *N* = 437 (Backfired *n* = 163; Mixed Feelings *n* = 179; Buffered *n* = 95). ** *p* < 0.01, *** *p* < 0.001. Pairwise comparisons were conducted following significant omnibus one-way ANOVAs. Reported *p* values are Tukey-adjusted for multiple comparisons. Cohen’s *d* reflects standardized mean differences between clusters. Positive difference scores indicate higher values for the first-listed cluster.

**Table A12:** Gratitude Letters Coding Theme and Word Count—Cluster Pairwise Comparison Statistics.

Coding Theme	Contrast	Diff	*p* Adj	Cohen’s *d*
Writer effort	Buffered-Backfired	−0.04	0.977	−0.03
Writer effort	Mixed Feelings-Backfired	0.44	0.037 *	0.38
Writer effort	Mixed Feelings-Buffered	0.48	0.029 *	0.42
Level of detail	Buffered-Backfired	0.00	1	0.00
Level of detail	Mixed Feelings-Backfired	0.51	0.007 **	0.47
Level of detail	Mixed Feelings-Buffered	0.51	0.011 *	0.47
Heartfelt sincerity	Buffered-Backfired	0.03	0.978	0.03
Heartfelt sincerity	Mixed Feelings-Backfired	0.38	0.039 *	0.38
Heartfelt sincerity	Mixed Feelings-Buffered	0.35	0.087	0.34
Reflection depth	Buffered-Backfired	−0.02	0.997	−0.01
Reflection depth	Mixed Feelings-Backfired	0.48	0.017 *	0.42
Reflection depth	Mixed Feelings-Buffered	0.50	0.020 *	0.44
Genuineness	Buffered-Backfired	−0.03	0.989	−0.02
Genuineness	Mixed Feelings-Backfired	0.44	0.021 *	0.41
Genuineness	Mixed Feelings-Buffered	0.47	0.020 *	0.44
Superficiality	Buffered-Backfired	−0.18	0.547	−0.18
Superficiality	Mixed Feelings-Backfired	−0.50	0.003 **	−0.50
Superficiality	Mixed Feelings-Buffered	−0.32	0.109	−0.33
Benefactor effort	Buffered-Backfired	−0.19	0.515	−0.19
Benefactor effort	Mixed Feelings-Backfired	0.30	0.147	0.29
Benefactor effort	Mixed Feelings-Buffered	0.49	0.010 *	0.48
Number of Words in Letter	Buffered-Backfired	−7.56	0.834	−0.10
Number of Words in Letter	Mixed Feelings-Backfired	20.96	0.185	0.27
Number of Words in Letter	Mixed Feelings-Buffered	28.52	0.061	0.37

Note. Total *N* = 236 (Backfired *n* = 76, Mixed Feelings *n* = 96, Buffered *n* = 64). * *p* < 0.05, ** *p* < 0.01. Pairwise comparisons were conducted following significant omnibus one-way ANOVAs. Reported *p* values are Tukey-adjusted for multiple comparisons. Cohen’s *d* reflects standardized mean differences between clusters. Positive difference scores indicate higher values for the first-listed cluster.
