# Supplementary material for: Individual Differences in the Affective Experience of Writing a Gratitude Letter: Who Benefits Most?
Source: Behav Sci (Basel). 2026 Feb 5;16(2):232. doi: 10.3390/bs16020232 (PMC12937751; doi:10.3390/bs16020232)
Supplement: Supplementary file 1 [file behavsci-16-00232-s001.zip › Supplementary Materials C- Vannoy et al., 2025. Gratitude Letter Coding Codebook.pdf]

Individual Differences in the Affective Experience of Writing a Gratitude Letter: Who Benefits Most?

Tanya K. Vannoy [1,*], Lisa C. Walsh [2], Luke Liao [1], and Sonja Lyubomirsky [1]
[1] University of California, Riverside, Riverside, CA 92521, USA
[2] Nanyang Technological University, Singapore 639798, Singapore
* Correspondence: tanya.vannoy@email.ucr.edu

Supplementary Materials: Gratitude Letter Coding Codebook

This document provides the finalized codebook used to code gratitude letters in the present study. All human coders and the AI-assisted coder applied the same definitions and scale anchors. Each theme was coded on a 5-point scale. Higher scores indicate greater presence of the construct.

Writer Effort

Definition:
Extent to which the letter reflects time, care, and effort by the writer, based on length and elaboration.

Coding Instructions:
5 = The letter writer seems to have put a lot of effort into the letter (wrote a longer letter, provided a lot of details)
1 = The letter writer did not seem to put any effort writing the letter (wrote a shorter letter, did not include many details, or did not seem to take the intervention seriously)

Scale:
1 = No effort at all
2
3
4
5 = A great deal of effort

Level of Detail

Definition:
Degree of specificity provided when explaining reasons for gratitude (e.g., context, anecdotes, examples).

Coding Instructions:
5 = The letter writer provided specific details about why they are grateful (e.g., specific context, specific anecdotes, expanded with specific examples, not just general examples). Specificity is key. You should be able to see the story through the writer's eyes and be transported to the story the writer is conveying (e.g., a specific time and place).
1 = The letter writer didn't provide specific details or specific examples about the reason they are thankful.

Scale:
1 = Not at all detailed
2
3
4
5 = Extremely detailed

Heartfelt Sincerity
Definition:
Extent to which the letter expresses genuine feelings about what it meant to receive support from the benefactor.

Coding Instructions:
5 = The letter seems heartfelt and sincere (e.g., the writer's language describes what it has meant to receive support from the benefactor; letter mentions how specific situation(s) made them feel). In other words, the letter writer is showing some vulnerability (i.e., lowering their emotional barriers) and showing warmth in their letter.
1 = The letter does not seem heartful and sincere. In other words, the language used is not specific about what it has meant to receive support from the benefactor, or little emotionality is displayed, or the letter is written using language that indicates the participant was not taking the activity seriously.

Scale:
1 = Not heartfelt or sincere at all
2
3
4
5 = Extremely heartfelt and sincere

Reflection Depth
Definition:
Degree to which the writer demonstrates thoughtful reflection on the benefactor's actions and their significance.

Coding Instructions:
5 = The letter writer seems to have reflected deeply about their benefactor's action. In other words, it is evident that a lot of thought went into writing the letter. The author uses explicit language, particularly when recalling what the kind act has meant to them beyond simply stating that "it meant a lot."
1 = The letter writer seems to have not reflected deeply about their benefactor's action (i.e., you can feel that the reflection was surface level and did not require deep thought), particularly when recalling what the kind act has meant to them (i.e., writer only explains that it has meant a lot, but don't go into detail).

Scale:
1 = No depth of reflection at all

2
3
4
5 = A great deal of depth of reflection

Genuineness
Definition:
Extent to which the letter conveys authentic gratitude.

Coding Instructions:
5 = The gratitude reflected in the letter seemed genuine (i.e., letter showed that they were truly grateful to their benefactor). In other words, it seems that the letter writer made a sincere effort to write a letter that was detailed, heartfelt and sincere and that provided depth of reflection.
1 = The gratitude reflected in the letter did not seem genuine

Scale:
1 = Not at all genuine
2
3
4
5 = Extremely genuine

Superficiality
Definition:
Degree to which the letter lacks substance and reads as generic or formulaic.

Coding Instructions:
5 = The letter has a lot of emotional and sentimental language but seems less personal and has no substance. In other words, the letter seems like a “Hallmark card.”
1 = The letter seems very personal and authentic/genuine. A personalized letter written from the heart.

Scale:
1 = Not at all superficial
2
3
4
5 = Extremely superficial

Benefactor Effort
Definition: Extent of effort implied by the benefit provided, based on the reason the writer expressed gratitude.

Coding Instructions:
5 = Look at the content of the letter wholistically and put yourself in the shoes of the person doing the kind act. Does it feel like a heavy lift? If so, code 4-5, depending on the additional

context. In other words, the kind act that the Actor is grateful for seems to have taken a great deal of effort on the part of the Target (i.e., the letter recipient). This could be something that took a considerable amount of time or seemed like a heavy lift. The kind act can be tangible (e.g., helping pay a significant amount of money like the costs of 4 years of college) or intangible (e.g., consistently providing support through a divorce or illness).
3 = The kind act that the Actor is grateful for seems to have taken a medium amount of effort (e.g., help moving, help paying lower amount bills (in the hundreds or lower thousands of dollars)
1 = The kind act that the Actor is grateful for seems to have taken a low amount of effort or the kind act is not specifically mentioned in the letter

NOTE: If the target is a parent, score their level of effort pretending they are not a parent. In other words, don't give them less points because they are just doing what a "parent" should do. If what the parent is doing requires a "heavy lift" or a significant amount of time (e.g., attending or driving their kids to all sports events, putting work aside to help their child through a difficult life circumstance), you can score this as a 3, 4 or 5 depending on the additional context of the letter.

Scale:
1 = No effort at all or effort not specified
2
3
4
5 = A great deal of effort
