# Supplementary material for: Individual Differences in the Affective Experience of Writing a Gratitude Letter: Who Benefits Most?
Source: Behav Sci (Basel). 2026 Feb 5;16(2):232. doi: 10.3390/bs16020232 (PMC12937751; doi:10.3390/bs16020232)
Supplement: Supplementary file 1 [file behavsci-16-00232-s001.zip › Supplementary Materials A - Vannoy et al., 2025. Links to Original Studies and Materials.pdf]

Individual Differences in the Affective Experience of Writing a Gratitude Letter: Who Benefits Most?

Tanya K. Vannoy [1,*], Lisa C. Walsh [2], Luke Liao [1], and Sonja Lyubomirsky [1]
[1] University of California, Riverside, Riverside, CA 92521, USA
[2] Nanyang Technological University, Singapore 639798, Singapore
* Correspondence: tanya.vannoy@email.ucr.edu

Original Materials and Datasets

Materials and datasets from the original studies (e.g., surveys, consent forms, and datasets) can be found:

1. Regan, Walsh, Lyubomirsky (2022) - Are some ways of expressing gratitude more beneficial than others? results from a randomized controlled experiment
    a. Article: https://doi.org/10.1007/s42761-022-00160-3
    b. Materials: https://osf.io/rp34a/overview?view_only=9857064e8d2544c69889f9908ce14c12

2. Walsh, Regan, Twenge, Lyubomirsky (2022) - What is the optimal way to give thanks? Comparing the effects of gratitude expressed privately, one-to-one via text, or publicly on social media
    a. Article: https://doi.org/10.1007/s42761-022-00150-5
    b. Materials: https://osf.io/4bwnf/overview

3. Walsh, Regan, Lyubomirsky (2022) - The role of actors, targets, and witnesses: Examining gratitude exchanges in a social context
    a. Article: https://doi.org/10.1080/17439760.2021.1991449
    b. Materials: https://osf.io/xj6s9
